# Prophylactic Effects of Quinine

**Published:** 1864-06

**Authors:** Samuel Logan

**Affiliations:** P. A. C. S.


### CONFEDERATE STATES MEDICAL & SURGICAL JOURNAL.

EXPERIENTIA DOCET.

Vol. I. | RICHMOND, JUNE, 1864. | No. 6.

### ORIGINAL COMMUNICATIONS.

Art. I.—*Prophylactic Effects of Quinine.* By Samuel Logan, P. A C. S.

The following table was compiled with the view of enabling the writer to arrive at some definite conclusion as to the prophylactic power of quinine. The items were collected, either by himself or the medical officer in charge, to whose cheerful co-operation he is much indebted. It will be observed that in no single camp was the agent unanimously adopted; in many, indeed, its use was resorted to by a minority only. So far as my object was concerned, this was rather fortunate than otherwise, as it has enabled me to *compare* the results among *those situated under the same identical circumstances* in all particulars, *except the use or neglect of the agent* whose effect we are investigating. I would call attention to the fact, that much more importance should be attached to the results developed under these circumstances, than when the experiments are made with separate corps, *all* of whom either took or neglected to take the quinine as a prophylactic. I know of no statistical researches, except of my limited extent, in which this peculiarity obtains—in fact, our statistical knowledge on the subject is extremely limited—and I therefore deem it of some importance to invite the attention of the profession to the table I have collected. All of the troops from whom these items have been gathered, were stationed in the most highly malarious regions in the Confederacy.

With the exception of a few favored localities, the whole country has to be abandoned by its white inhabitants as the summer approaches. They usually resort to the pine lands or the sea-shore about the first of May, and return about the first of November.

The peculiar typography of our Southern Atlantic lowlands is well known, but may be briefly mentioned. The coast line is indented with numerous inlets, bays and salt-water creeks, which form a complicated network extending from ten to twenty miles from the sea-shore proper. These creeks and estuaries are generally fringed with wide plains of salt-marshes, except when the larger rivers freshen them and form the valuable fertile rice lands. The islands formed by this network of water courses are, with the exception of the marshes, composed of a light sandy soil, in which the stratum of clay lies from three to six or eight feet below the surface. They were devoted to the culture of the famous "sea-island" or long staple cotton. As we come more towards the interior, and especially near to the larger streams, this light sea-island soil more or less disappears, and we have level tracks of a stiffer earth, [illegible]

ally sandy, but in most cases having a stratum of red clay, either near or quite up to the surface. The undergrowth is here extremely luxuriant, and reminds one of the pictures of African jungles. The country is intersected with swamps, some of them cultivated in rice, and presenting a considerable alluvial deposit upon a bed of blue clay. Next to this belt of "low-lands," we come to the *pine* region; a dry and porous sandy soil, with clay at a considerable depth, the sand in the higher localities being of a bright yellow or white, and in the lower ones dark grey. This flat belt of "pine barrens" gradually changes into the rolling country of the interior, and so on up to the mountain slopes.

The malarial fevers prevail throughout the sea-islands, with a few exceptions; through the whole of the belt of level low-lands, and to a considerable extent in the lower of the pine lands. The higher portions of the latter, and certain favorable localities along the sea-shore, and among the sea-islands, are more or less exempt, and are resorted to during the summer months. The enemy have occupied almost all of the healthy sea-shore, while our lines of defence extend through the sickly low-lands, just within the belt of sea-islands. In order to promote the health of the troops, such of them as are located along the lines in the neighborhood of healthy pine land resorts are moved in the summer months to such places. Those commands, however, who do the picket duty, even though their summer camps be pitched in a healthy pine-land village, are still subjected to malarial influences while on this out-post duty along the line, day and night, at an average of about one-third or one-fourth of the time. Those commands who were, as a general rule, retained during the summer in their healthy resorts, were not provided with quinine to use as a prophylactic, and have not therefore been made the subjects of investigation. They had little or no fever among them; while those whom it was necessary, for strategical purposes, to retain in the unhealthy country, and the cavalry who did the picket duty in sickly localities were provided; and a reference to the table will show to what an extent each command availed itself of the agent, and what were the results.

In some cases the quinine was taken in the morning, in others at night, four grains being the quantity used. Some of the medical officers believed before-hand in its efficacy; others held their opinions in suspense, while a few disbelieved in its use as a prophylactic. I was among those who were not inclined to form a definite opinion until a larger mass of evidence had been collected in such a manner as to show the *comparative* results among those placed under identical circumstances, in all particulars, excepting the use of the agent. It will be well to make a few remarks concerning the different corps and their precise localities.

1st. *The Rutledge Mounted Riflemen and Horse Artillery* did picket duty along the line between the Coosauhatchie and the Conobahie rivers, a section of country of an undoubted malarious character. They were camped in a tolerably healthy pine land village, McPhersonville, three and a half miles from the Pocotaliga station, on the C. and S. R. R.

2d. *Captain Earle's Battery* was stationed during the summer of 1862, on James Island, near Charleston S. C (a location of notoriously bad reputation for health) until September, when it was moved to a position about six miles below the Hardeeville station, on the C. and S. R. R., not far from the Savannah river. They gained nothing by the change, so far as a healthy camp was concerned. The next summer they were stationed at and near the Green Pond station, between the Conobahee and Ashepoo rivers. This, also, is a highly malarious location.

3d. *Company I, Third S. C. C.*, as I am informed by Dr. W. M. Bailey who has charge of them, was "stationed in the interior of Wadmalaw Island, a situation, from the evidence of the physician who practised there before the war, exceedingly unhealthy as regards fevers, in the midst of swampy lands and numerous stagnant ponds."

About four grains of quinine was given each day at "Retreat" in which quantity it was administered from the 1st July until the 1st October.

Dr. Bailey goes on to say that not being supplied with a sufficient amount of the article he continued, from that date, to administer it in reduced doses of two grains until about the 15th October. "Whether from the effect of climate, or from the reduced quantity given, I had at this time many more cases of fever."

4th. *Company B, 6th regiment, S. C. C.*, was stationed near the river bank of the Edisto, a situation personally known to me as very unhealthy. With them also the quinine was reduced from October 1st, from four to two grains, and stopped on the 15th.

5th. *Company D, 5th regiment, S. C. C.*, arrived at its camp near the Conobahee river on the 27th September, having been stationed in Charleston during the midsummer. The men were exposed on picket in a very malarious country, at the most unhealthy season, but Captain Z. Davis, in command, immediately commenced to issue "four grains of quinine in a wine glass of whiskey" every morning at reveille, and continued it until after frost.

6th. *Blake's Battery* was stationed during the whole summer on James Island. They moved camp three times, but were always located in positions which Assistant Surgeon Mallory C. Rivers in charge—who has kindly furnished me with the details—knows to have been unhealthy, from his intimate acquaintance with the facts before the war. The quantity of quinine issued was, as in the other cases, four grains daily.

7th. *Kirk's Partizan Rangers,* and the five companies of the 4th S. C. C., were situated in all respects as the Rutledge Mounted Riflemen, and had the same duty to perform. They also took the same quantity of quinine.

8th. *The Washingtsn Artillery* spent the summer in a very malarious location, amid the swamps between the Edisto and Ashepoo rivers. Assistant Surgeon W. S. Canneu, writes that "on the 1st of July, 1863, the administration of quinine was commenced, in four grain doses every night and continued until the 12th of November."

I have only to remark, in conclusion, that in no case has any cumulative effect been observed to follow the continued use of the quinine.

It would seem from these statistics that, though not an absolute prophylactic, the degree of protective power possessed by the agent fully warrants its use. If four-fifths of the fever cases are prevented, it should surely be used. It may be well to explain that under the head of "*number who took quinine irregularly*" are included those who would forget or neglect to take it some three or four days in the week, or take it one day and forget it the next, or omit it for a week at a time.

CONSOLIDATED TABLE OF CASES.

Total number who took no quinine, 230; had fever, 134; ratio per 1,000 of fever cases to patients, 582.60, or 1 in every 1.71 patients; ratio per 1,000 of severe cases to total cases, 313.43, or 1 in every 319 cases.

Total number who took quinine irregularly, 246; had fever, 96; ratio per 1,000 of fever cases to patients, 390.24, or 1 in every 2 56 patients; ratio per 1,000 of severe cases to total cases, 291.66, or 1 in every 3.71 cases.

Total number who took quinine regularly, 506; had fever, 98; ratio per 1,000 of fever cases to patients, 193.67, or 1 in every 5.16 patients; ratio per 1,000 of severe cases to total cases, 326,53, or 1 in every 3.06 cases.

